# Emergence of tick-borne diseases at northern latitudes in Europe: a comparative approach

**DOI:** 10.1038/s41598-017-15742-6

**Published:** 2017-11-24

**Authors:** Atle Mysterud, Solveig Jore, Olav Østerås, Hildegunn Viljugrein

**Affiliations:** 1 Centre for Ecological and Evolutionary Synthesis (CEES), Department of Biosciences, University of Oslo, P.O. Box 1066 Blindern, NO-0316 Oslo, Norway; 2 Department of Food, Water, Zoonotic & Vector-borne Infections, The Norwegian Institute for Public Health, P.O. Box 4404 Nydalen, NO-0403 Oslo, Norway; 3 Department of the Norwegian Cattle Health Services, TINE Norwegian Dairies BA, Oslo, NO-1431 Ås Norway; 4 Norwegian Veterinary Institute, P.O. Box 750 Sentrum, NO-0106 Oslo, Norway

## Abstract

The factors that drive the emergence of vector-borne diseases are difficult to identify due to the complexity of the pathogen-vector-host triad. We used a novel comparative approach to analyse four long-term datasets (1995–2015) on the incidence of tick-borne diseases in humans and livestock (Lyme disease, anaplasmosis and babesiosis) over a geographic area that covered the whole of Norway. This approach allowed us to separate general (shared vector) and specific (pathogen reservoir host) limiting factors of tick-borne diseases, as well as the role of exposure (shared and non-shared pathogens in different hosts). We found broadly similar patterns of emergence across the four tick-borne diseases. Following initial increases during the first decade of the time series, the numbers of cases peaked at slightly different years and then stabilized or declined in the most recent years. Contrasting spatial patterns of disease incidence were consistent with exposure to ticks being an important factor influencing disease incidence in livestock. Uncertainty regarding the reservoir host(s) of the pathogens causing anaplasmosis and babesiosis prevented a firm conclusion regarding the role of the reservoir host-pathogen distribution. Our study shows that the emergence of tick-borne diseases at northern latitudes is linked to the shared tick vector and that variation in host-pathogen distribution and exposure causes considerable variation in emergence.

## Introduction

Many emerging infectious diseases are of wildlife origin, are vector-borne^1,2^, and present a growing health concern for both humans and livestock^3,4^. Lyme disease (or Lyme borreliosis) is considered the archetype of an emerging infectious disease^5^, and it is currently the most common zoonotic vector-borne disease at northern latitudes. The ticks *Ixodes ricinus* in Europe and *I. scapularis* in North America are the main vectors for the spirochete bacteria of the *Borrelia burgdorferi* sensu lato (s.l.) complex, which are the causative agents of Lyme disease^6,7^. The distribution of ticks continues to expand northwards in latitude and upwards in elevation in both Europe^8^ and North America^9^. Climate change is one driver of this expansion in the distribution of *Ixodes* ticks^8,10–12^. Other drivers of Lyme disease emergence, such as land use, which causes habitat changes and subsequent changes in host diversity and abundance^13^, are clearly important, although their detailed roles are debated^14–17^. Distinguishing the relative importance of different limiting factors of the same disease is particularly difficult for complex multi-host systems such as the one underlying Lyme disease^18^. However, these *Ixodes* ticks transmit a variety of other pathogens with different transmission cycles^19^, allowing for comparative approaches to be used to identify the relative importance of different drivers.

**Figure 1:**
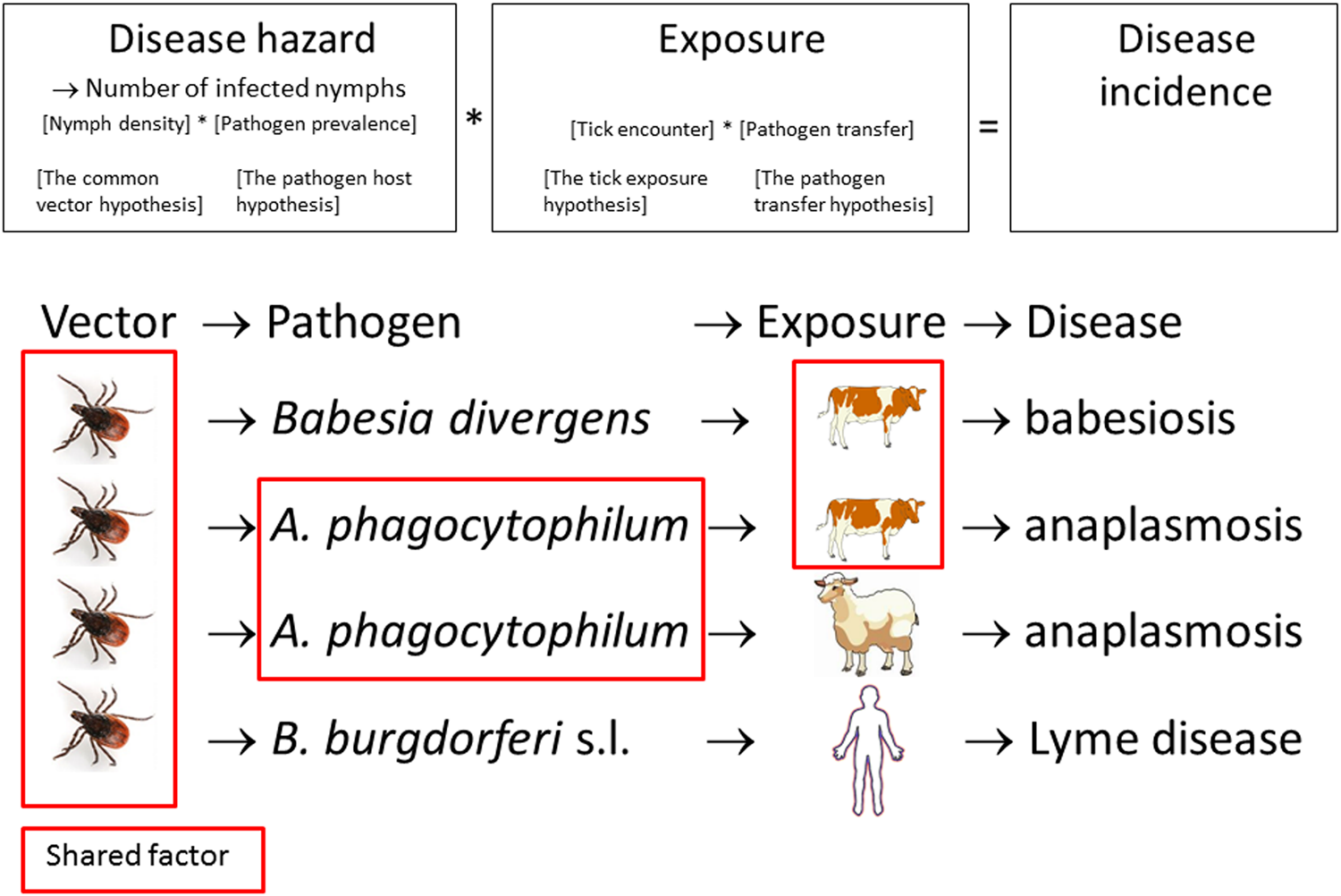
A conceptual overview of the comparative approach that enables the identification of general and specific limiting factors for tick-borne diseases. Underlying shared factors can cause synchrony in disease emergence, whereas restricted synchrony may reflect shared pathogens or similar exposure. (The tick, sheep, cow and human are Windows Clip Art. Used with permission from Microsoft).

Two other important tick-borne pathogens in Europe are the bacterium *Anaplasma phagocytophilum*, which causes tick-borne fever in sheep and cattle^20^, and the protozoan *Babesia divergens*, which causes babesiosis in cattle^21^. Both pathogens can also cause human disease^21,22^. The emergence of these different tick-borne diseases may in part reflect a common role of environmental changes affecting the tick vector^23^. However, the reservoir hosts differ among these three tick-borne pathogens. Thus, depending on the distribution of the reservoir host and whether the host populations of specific tick-borne pathogens are increasing or decreasing under environmental change, the emergence of different tick-borne diseases may vary in space and time. Thus, there may be ‘general’ drivers of tick-borne diseases that operate directly through the shared tick vector, as well as ‘specific’ drivers that are associated with the dynamics and distributions of the various reservoir hosts of the pathogen. In Europe, the most common pathogen that causes Lyme disease is *Borrelia afzelii*, which has a small mammal reservoir^24–27^; less common are *B. garinii* (in birds) and *B. burgdorferi* sensu stricto (a generalist). *A. phagocytophilum* has a wide range of reservoir hosts in Europe including wild ruminants, rodents, insectivores, birds, carnivores, and domestic animals^28^. There are at least four strains of *A. phagocytophilum* circulating in different reservoir hosts, and there is uncertainty regarding strain pathogenicity and transmission cycles. For the strain of *A. phagocytophilum* that infects livestock, the main wildlife reservoir is the red deer (*Cervus elaphus*)^29–31^. The reservoir of *B. divergens* includes cattle, and possibly deer^21,32^. A comparative approach enables the identification of the ‘general’ and ‘specific’ factors that drive the emergence of tick-borne disease (Fig. 1). Land use, such as the grazing of sheep and cattle, also indirectly affects disease incidence by influencing exposure to ticks. We mainly interpret exposure as the encounter rate or contact rate with ticks, while the pathogen transfer process may also be important in some cases (Fig. 1). By analysing both the numbers of cases and incidence, we can determine the effects of human exposure and livestock exposure, as well as the drivers of emergence. Land use practices are changing across much of Europe due to new environmental policies and an increase in more intensive livestock production, resulting in the increased use of infield grazing (milk-producing cows) and outfield pastures (heifers)^21^. Therefore, understanding the drivers of tick-borne disease emergence has important economic implications.

We use a unique comparative approach to analyse the long-term epidemiology (1995–2015) of three different tick-borne diseases (Lyme disease, anaplasmosis and babesiosis) and of the same disease (anaplasmosis) in two species of livestock (cattle and sheep). We use four datasets of long-term disease records with broad spatial extent, covering the whole of Norway, to assess 1) whether the diseases are emerging (as defined in ref.^33^) at a national or regional level, 2) whether emergence within regions is due to temporal or spatial increase, and 3) whether drivers are general across diseases or specific to each disease. If tick-borne diseases are limited by the vector population, with emergence associated with the distribution expansion of the vector *I. ricinus*, which is common to all diseases (*the common vector hypothesis*), we would predict high spatial and temporal synchrony in the emergence of tick-borne diseases (Table 1). It is also possible that emergence and/or annual variation are more strongly influenced by the pathogen-reservoir host interface (*the pathogen-host hypothesis*). The small mammal reservoirs of the Lyme disease pathogen are distributed across the whole country and predict no specific spatial pattern. Anaplasmosis is predicted to follow the geographic distribution of red deer, which is primarily restricted to the west coast of Norway. Babesiosis is connected to the cattle themselves and possibly to deer populations in general; hence no clear prediction can be made. Indeed, the uncertainty regarding the most quantitatively important reservoir hosts of both *A. phagocytophilum* and *B. divergens* are currently a limitation for making more specific predictions (see discussion for detail). Lastly, differences in exposure (*the tick exposure hypothesis*) should result in a more similar incidence of the cattle diseases (Fig. 1, Table 1).

**Table 1:** An overview of hypotheses and predictions for the temporal and spatial pattern of incidence across different tick-borne diseases, and the level of support based analysis of incidences of Lyme disease, babesiosis and anaplasmosis in cattle and anaplasmosis in sheep in Norway, 1995–2015.

Hypotheses	Rationale	Predictions	Support
The common vector hypothesis	If disease incidence is limited by the vector, we expect incidence to be linked to tick distribution	Shared trend over time across all diseases	+
		Common disease drivers (climate, deer populations)	+
		Similar spatial occurrence across all diseases	−
		Annual synchrony across all diseases	−
The pathogen-host hypothesis	If disease incidence is limited by the presence of the pathogen, we expect incidence to be linked to the reservoir host distribution	Similar spatial pattern of incidence in anaplasmosis in sheep and cattle	−
		Annual synchrony of incidence of anaplasmosis in sheep and cattle	−
		More anaplasmosis in areas with red deer	+
The tick exposure hypothesis	If disease incidence is limited by exposure, we expect disease incidence linked to land use practices affecting exposure	Similar spatial pattern of incidence in the two cattle diseases	+
		Shared trend over time for the cattle diseases	(+)
		Annual synchrony of cattle diseases	(+)

We documented the emergence of all the tick-borne diseases at the national level, consistent with the pattern expected with a common driver operating through the tick vector. The emergence of all diseases had both a temporal component (increased local incidence) and a spatial component (increased range) within regions. However, the low level of spatial correlation and annual synchrony across diseases also suggest the importance of disease-specific host-pathogen interactions and exposure in determining the epidemiology of these tick-borne diseases (Fig. 1). Furthermore, following initial increases during the first decade of the time series (1995–2005), the numbers of cases peaked at slightly different years and then stabilized or declined in the most recent years.

## Results

### Temporal and spatial emergence, synchrony and spatial extent

**Figure 2:**
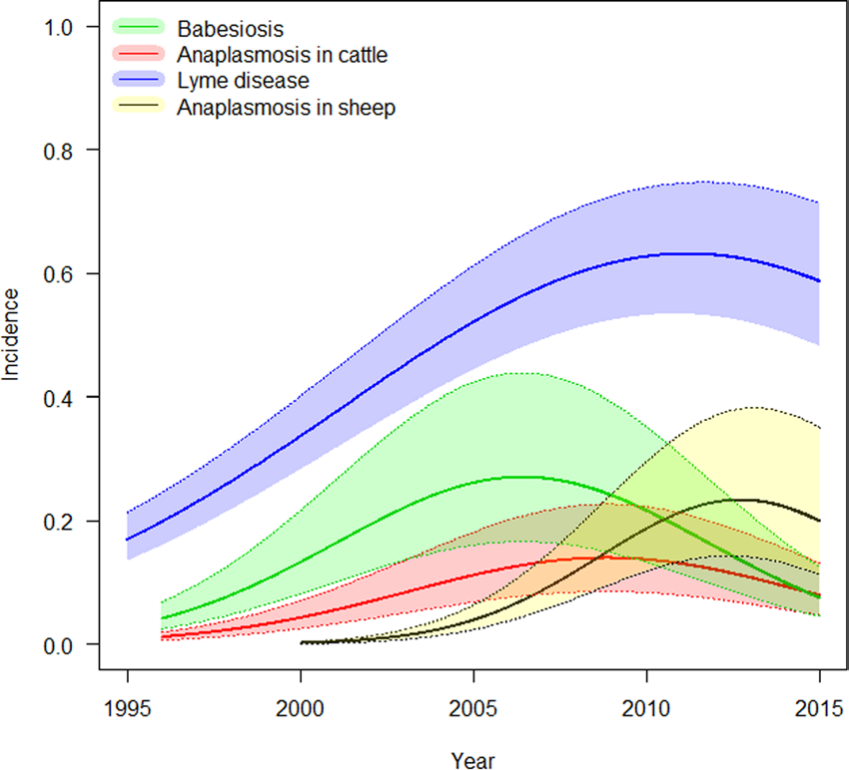
Emergence of tick-borne diseases in Norway from 1995 to 2015. Patterns of disease incidence over time for four tick-borne diseases: Lyme disease in humans, anaplasmosis in sheep, anaplasmosis in cattle, and babesiosis in cattle. Lines are predictions (±SE) from mixed-effects models with negative binomial errors and the units are the incidence per 10000 inhabitants for Lyme disease, per 500 outfield-grazing cattle for babesiosis in cattle, per 500 outfield-grazing cattle for anaplasmosis in cattle, and per 1000 sheep in the health data register for anaplasmosis in sheep. All of the lines are for the western region of Norway.

**Figure 3:**
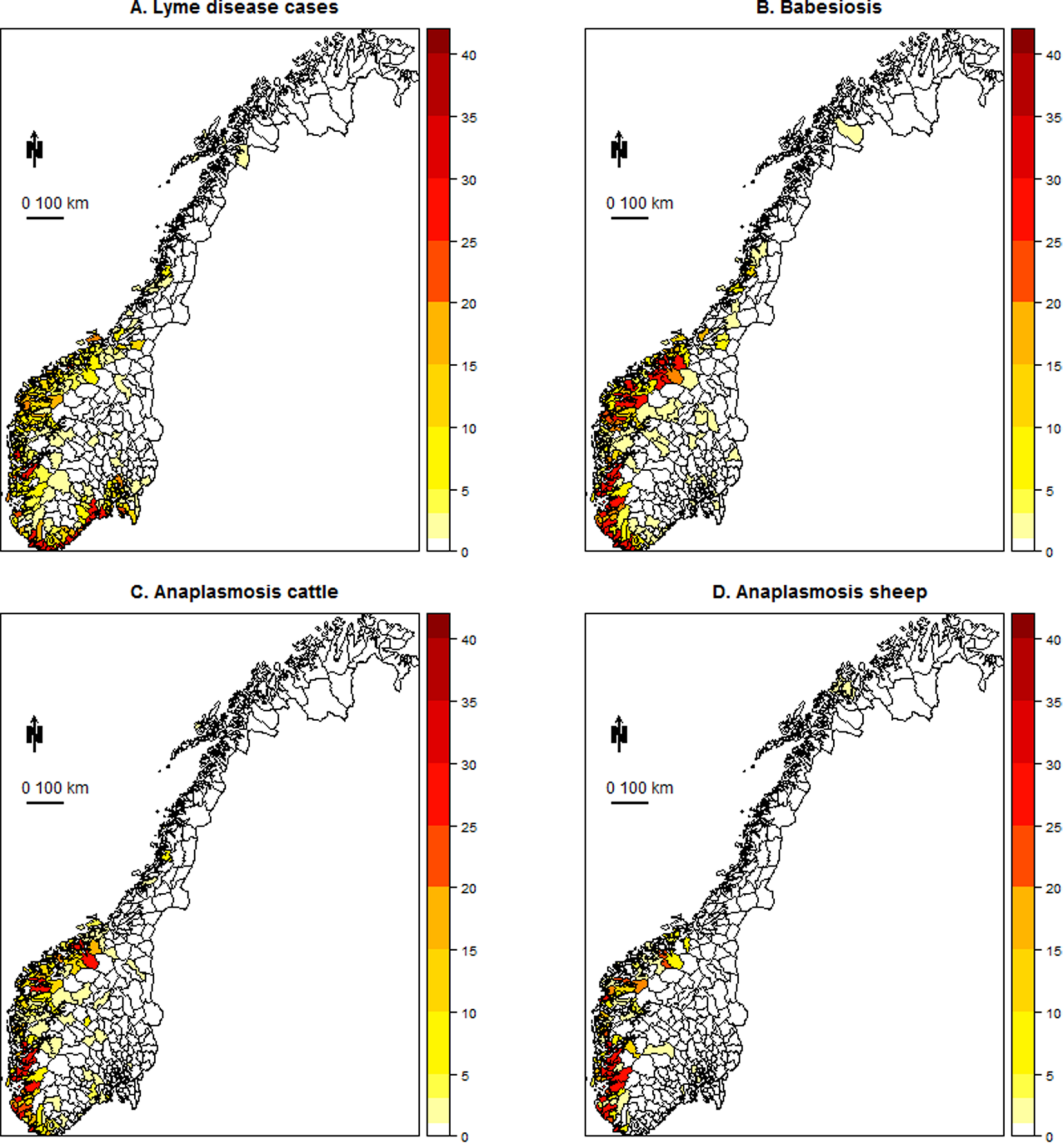
Spatial pattern of tick-borne diseases in Norway. Pattern of summed disease cases over the time period of 1995–2015 for 4 tick-borne diseases: Lyme disease in humans, anaplasmosis in sheep, anaplasmosis in cattle, and babesiosis in cattle. The map was created using several R packages (sp, rgdal, maptools, grid, and lattice) in R version 3.3.3. The shape-files with borders of municipalities are freely available from the Norwegian Mapping Authority (http://www.kartverket.no/en/data/Open-and-Free-geospatial-data-from-Norway/).

Lyme disease in humans, anaplasmosis in sheep and cattle, and babesiosis in cattle showed significant emergence over the studied time period (Fig. 2, Table 2, Supplementary Tables S1–S4). The disease records were analysed both in terms of disease incidence (number of cases for a given population size in a given area) and as disease occurrence (defined as municipalities with at least one disease case). The incidences and occurrences were analysed using negative binomial mixed-effects model and mixed-effects logistic regression models, respectively. Disease emergence was due to an increased disease incidence (the “year” term when analysing counts in a negative binomial mixed-effects model) and to an increased spatial occurrence (the “year” term in the mixed-effects logistic regression model, Table 2). For all diseases, disease incidence increased rapidly from 1995 onwards and then stabilized at some point after 2005 (Fig. 2). The pattern was similar when incidence was evaluated at the municipality level (Fig. 2) and when the total incidence or number of cases summed for all municipalities annually in all of Norway (Supplementary Fig. S1) was used. The spatial occurrence increased markedly over the time period for all diseases (Supplementary Fig. S2). Lyme disease was found in 52.6% of the 428 municipalities of Norway, revealing the presence of the tick vector across large areas (Fig. 3). The spatial distributions of the other diseases were more restricted (Fig. 3). Anaplasmosis in sheep was found in only 17.9% of municipalities (among the 402 municipalities with at least 1 sheep), whereas anaplasmosis and babesiosis in cattle were found in 30.2% and 34.3% of municipalities, respectively (among the 420 municipalities with at least 1 cow). There was a high spatial correlation in mean incidence only between anaplasmosis and babesiosis in cattle (r_Pe_ = 0.73). There was a moderate spatial correlation in total disease cases for anaplasmosis in sheep and babesiosis (r_Pe_ = 0.49) and anaplasmosis in cattle (r_Pe_ = 0.48), and a low spatial correlation between the number of cases or incidence of Lyme disease and the livestock diseases (r_Pe_ ≤ 0.26, Table 3). These spatial patterns are consistent with tick exposure or reservoir host being the main driver of disease incidence, rather than the common vector. There was some evidence of year-to-year synchrony among the diseases when analysing the raw data summed annually at the country level. However, after adjusting for the main spatial structure and the main trend over years (Fig. 2), there was no annual synchrony among the diseases in the residuals of the models.

**Table 2:** Models of tick-borne diseases. Parameter estimates of incidence from mixed-effects models using (**A**) counts in a negative binomial model and (**B**) presence or absence of disease in a logistic regression model for Lyme disease in humans, babesiosis in cattle, anaplasmosis in cattle, and anaplasmosis in sheep from the whole of Norway for the years 1995–2015. Random intercepts were 192 (**A**) and 253 (**B**) municipalities. Except for anaplasmosis in sheep (**A** and **B**) and anaplasmosis in cattle (**B**), random intercepts were nested in 10 counties.

A. Negative binomial model	Lyme disease in humans		babesiosis in cattle		anaplasmosis in cattle		anaplasmosis in sheep	
	Estimate	SE	Estimate	SE	Estimate	SE	Estimate	SE
Intercept	−10.171	0.155	−7.975	0.483	−8.749	0.484	−13.544	0.675
year	**0.431**	**0.053**	**0.283**	**0.040**	**0.674**	**0.059**	**1.618**	**0.181**
year^2^	*−0.205*	*0.044*	*−0.579*	*0.043*	*−0.505*	*0.053*	*−0.692*	*0.130*
log(mean spatial deer density + 0.01)	**0.441**	**0.075**	**0.882**	**0.135**	**0.572**	**0.136**	**1.010**	**0.256**
temporal deer density	**0.108**	**0.042**						
NAO-DJF (lag 0 or 1 yr)	**0.072**	**0.023**			**0.072**	**0.036**		
NAO-MAM (lag 1 or 2 yr)	*−0.077*	*0.023*	*-0.072*	*0.030*				
sqrt(prop(human settlement))	*−0.460*	*0.058*	*-0.486*	*0.119*			*−0.445*	*0.204*
sqrt(distance to fjord)	*−0.322*	*0.094*						
North-UTM	*−0.593*	*0.143*					−1.816	0.351
sqrt(prop(agriculture))	*−0.315*	*0.077*						
Region «South» [Lyme] or «West» [anaplasmosis]	**0.239**	**0.353**					**3.841**	**0.623**
prop(area >200 m a.s.l.)	*−0.366*	*0.095*						
sqrt(temporal autocorrelation)			**0.198**	**0.032**	**0.129**	**0.037**		
Region «South» * year	*-0.381*	*0.070*						
Region «South» * year^2^	**0.144**	**0.061**						
Health recordings						**0.742**	**0.091**	
B. Binomial model								
Intercept	−2.454	0.201	−4.044	0.430	−4.798	0.265	−9.113	0.812
year	**0.397**	**0.047**	**0.253**	**0.053**	**0.536**	**0.065**	**0.807**	**0.102**
log(mean no. susceptible)	**0.820**	**0.075**	**1.149**	**0.127**	**0.983**	**0.130**	**1.648**	**0.240**
log(mean spatial deer density + 0.01)	**0.615**	**0.126**	**0.709**	**0.171**	**0.513**	**0.181**	**1.348**	**0.305**
North-UTM	*−1.499*	*0.209*	*−1.050*	*0.351*	*−1.203*	*0.180*	*−2.429*	*0.397*
sqrt(distance to fjord)	*−0.960*	*0.120*	*−0.364*	*0.179*	*−0.391*	*0.189*		
Region «West»			**1.275**	**0.643**	**1.740**	**0.291**	**3.451**	**0.630**
Health recordings							**0.878**	**0.133**
sqrt(prop(agriculture))	*−0.309*	*0.089*						
year * log(mean no. humans)	**0.179**	**0.049**						
temporal autocorrelation > 0			**1.013**	**0.121**	**0.884**	**0.144**	**0.668**	**0.225**

Continuous variables are scaled to have a mean = 0 and a variance = 1. sqrt = square root-transformed; prop = proportion; temporal autocorrelation = incidence of disease in the previous year. Numbers in italics indicates a significant negative effect, whereas bold font indicates a significant positive effect. For z- and p-values, see appendix tables 1–4. Number of susceptible hosts refers to human population for Lyme disease, cattle population (+1) for babesiosis in cattle, cattle population (+1) for anaplasmosis in cattle, and sheep population for anaplasmosis in sheep.

**Table 3:** Spatial and temporal correlations of disease cases and incidence in Norway.

	Lyme disease	Babesiosis	Anaplasmosis cattle	Anaplasmosis sheep
**Spatial**				
Lyme disease		**0.24** [0.14, 0.42]	**0.23** [0.14, 0.36]	**0.26** [0.10, 0.43]
Babesiosis	**0.16** [0.06, 0.35]		**0.73** [0.41, 0.88]	**0.22** [0.12, 0.36]
Anaplasm. cattle	**0.14** [0.05, 0.28]	**0.79** [0.67, 0.89]		**0.21** [0.12, 0.35]
Anaplasm. sheep	0.06 [−0.01, 0.21]	**0.49** [0.29, 0.73]	**0.48** [0.28, 0.71]	
**Temporal**				
Lyme disease		−0.14 [−0.62, 0.37]	0.05 [−0.58, 0.58]	0.42 [−0.36, 0.85]
Babesiosis	−0.18 [−0.66, 0.33]		**0.54** [0.03, 0.81]	−0.02 [−0.31, 0.37]
Anaplasm. cattle	0.03 [−0.59, 0.56]	**0.49** [0.02, 0.78]		0.07 [−0.60, 0.49]
Anaplasm. sheep	0.41 [−0.33, 0.81]	0.03 [−0.28, 0.42]	0.03 [−0.60, 0.49]	

The spatial correlation (Pearson) between the number of cases (bottom-left) and mean incidence (top-right) between 4 tick-borne diseases: Lyme disease in humans, babesiosis in cattle, anaplasmosis in cattle, and anaplasmosis in sheep, averaged over the period 1995–2015. Confidence intervals were obtained by bootstrapping (using the 2.5 and 97.5 percentiles). Spatial correlations are based on data from each municipality from all of Norway, and temporal correlations are first-differenced series of total incidences/sums per year for all of Norway. Pearson correlations in bold have values with 95% confidence intervals that do not overlap zero.

### Drivers affecting spatial and temporal variation

All of the diseases declined towards the north, where the climate is colder, and 3 of the 4 diseases occurred most frequently along the coast, which has the climate where ticks thrive (Table 2). Babesiosis and anaplasmosis in cattle and anaplasmosis in sheep had the highest incidences along the west coast region of Norway. The incidences of all 4 diseases were higher in areas with denser deer populations (Table 2). For 3 of the 4 diseases, the incidence was lower in areas with a large proportion of human settlements. Disease incidence was also associated with the climate variable NAO: positively for the winter NAO index (wet, warm winters) for 2 of 4 diseases and negatively for the spring NAO index (warm springs) for 2 of 4 diseases. In addition, there were some disease-specific patterns. There were higher levels of Lyme disease incidence in the south, in areas with little agriculture and mainly low elevation areas, and the temporal increase in Lyme disease was lower in the southern region. In general, the patterns of incidence for anaplasmosis and babesiosis in cattle were more similar to each other than were the patterns for anaplasmosis in sheep and cattle, suggesting that tick exposure or the pathogen-reservoir host distribution is influencing incidence.

## Discussion

Our study is the first to quantitatively measure the emergence of several tick-borne diseases at the northern distribution limit of *I. ricinus* ticks in Europe. After a phase of initial increases in both the number of cases and incidence for all 4 diseases, the values stabilized (at slightly different times for each disease) in the period 2005–2010, remaining stable or decreasing slightly thereafter. The broadly similar patterns over time and space in the four tick-borne diseases are strong evidence of a shared driver associated with the tick vector population (supporting the common vector hypothesis, Fig. 1, Table 1). All of the diseases were more common in the coastal areas, mainly in the southern half of Norway, which has a favourable climate for ticks. However, there were large variations in the spatial extent and distribution of incidence among the different tick-borne diseases, as well as a lack of annual synchrony, suggesting the roles of specific drivers associated with reservoir host population dynamics and distribution (supporting the pathogen-host hypothesis, Fig. 1, Table 1). In addition, exposure was important, as emergence was highly correlated between babesiosis and anaplasmosis in cattle (supporting the tick exposure hypothesis), whereas anaplasmosis was only weakly correlated between sheep and cattle (not supporting the pathogen-host hypothesis, Fig. 1, Table 1).

A common role of environmental factors that operate via the tick vector, leading to an emergence of these diseases across Norway, is the most likely explanation for the observed broad pattern. Over the past decades, the distribution of *I. ricinus* ticks has expanded 400 km northwards and upwards in elevation in Norway^34^, and similar elevational and latitudinal increases have been reported in other locations in Europe^8^. All of the models explaining variation in disease incidence included proxies for climate that are associated with a well-known coastal-inland gradient. Global warming has resulted in a 2–3 week shortening of the winter season and an earlier and warmer spring in the coastal areas of Norway^35^, which may at least partly explain the synchronous disease emergence. A similar strong effect of climate operating via the tick vector was implied for Lyme disease emergence in Scotland^36^. Incidence for all of the diseases was also associated with high deer population density, as previously shown for Lyme disease^37^. The positive effect of high deer abundances on incidence of all diseases is likely due to the fact that deer are important reproduction hosts for adult ticks, and it therefore provides evidence for the common vector hypothesis (Table 1).

The more detailed temporal and spatial patterns point to specific drivers of each disease, which are presumably associated with reservoir host distribution and tick exposure. Recent changes in land use, causing habitat conversion, are key drivers of the changes in the mammalian host community and the re-emergence of Lyme disease in the USA^14,16^. In our study, the presumed main reservoir hosts of the 3 pathogens have widely different dynamics and distributions, which allowed us to at least partly distinguish the specific drivers associated with the temporal dynamics and spatial distribution of each host population. There are many competent small mammal reservoir hosts for *B. burgdorferi* s.l. in Europe, most importantly the wood mouse *Apodemus sylvaticus*, the bank vole *Myodes glareolus* and the common shrew *Sorex araneus* ^38^. As expected based on the wide geographic distributions of these reservoir hosts, the prevalence of *B. burgdorferi* s.l. in nymphal ticks is typically quite high (~20%) across the whole tick distribution of Norway^39–41^, suggesting that access to competent reservoir hosts is not limiting pathogen distribution. This situation can at least partly explain the very wide distribution of Lyme disease in Norway (53.5% of municipalities) compared to the more restricted distributions of anaplasmosis and babesiosis (17.9–35.6%).

There is more uncertainty regarding the reservoir hosts causing anaplasmosis and babesiosis, and it is therefore difficult to predict the disease distribution expected from the pathogen host hypothesis. *A. phagocytophilum* is consistently less prevalent than *B. burgdorferi* s.l. in questing ticks in Norway, as observed elsewhere in Europe^42,43^. The estimated prevalence of *A. phagocytophilum* in questing nymphs is typically less than 5% in the eastern, southern and northern parts of Norway^44–47^, whereas a prevalence of 8.8%^40^ to 16.7%^46^ was reported in western Norway. Hence, the higher incidence of anaplasmosis in sheep and cattle along the west coast probably reflects higher pathogen prevalence, in combination with higher density of ticks, together causing a higher density of infected nymphs. This prevalence might be associated with the distribution of red deer, which occur mainly in dense populations along the west coast of Norway (Supplementary Fig. S3). There are at least four strains of *A. phagocytophilum* circulating with different reservoir hosts, but there is still uncertainty regarding strain pathogenicity and transmission cycles. The strain of *A. phagocytophilum* having a rodent reservoir host is vectored by the nest-dwelling *I. trianguliceps* tick^30^. Hence, the rodent-borne strain is unlikely to come in contact with livestock or humans. Two of the strains are known to infect cervids^30^, and both roe deer (*Capreolus capreolus*) and red deer may have high infection levels^28^. Among the genovariants of *A. phagocytophilum* ^29,30^, the one circulating in red deer is the one most commonly found in livestock^48,49^. Several studies now show that the strain circulating in roe deer and livestock are different^30,31,50^. The spatial pattern of disease incidence we observed is consistent with this view of red deer rather than roe deer being the main wildlife reservoir of the *A. phagocytophilum* genovariant that causes disease in livestock. The red deer is currently expanding its distribution towards the south, east and north, which may lead to an increased risk of anaplasmosis in these regions as the red deer population sizes increase. However, as both sheep and cattle may also serve as reservoir hosts to *A. phagocytophilum* ^28,48^, it is difficult to determine the extent to which the wildlife reservoir hosts determine disease incidence. Further, as co-infection of *A. phagocytophilum* and *B. divergens* has been documented in cattle^51^, a firm separation of the pathogen-host hypothesis and tick exposure hypothesis is difficult.

*B. divergens* is the least prevalent pathogen among the three pathogens^52^. However, the extent of spatial and temporal variation in its distribution and its reservoir hosts are not well described^53^. Sera from cows along the coast of southern Norway revealed that 27% of cattle from farms in forested areas were seropositive for *B. divergens* ^54^. Cattle are the main reservoir for *B. divergens*. Though roe deer and red deer have also been implicated in Europe^32,55^, it is likely due to the difficulty to separate *B. divergens* from *B. capreoli* as their 18 S rDNA strains are quite similar^56^. It remains to be determined whether red deer play a role for both *A. phagocytophilum* and *B. divergens*, but current evidence suggests that it is unlikely^56^. Pathogens invading the same reservoir host may lead to co-infections and correlated disease emergence. In eastern North America, small mammals are competent reservoirs for the three pathogens *B. burgdorferi* s.l., *B. microti*, and *A. phagocytophilum* ^19^. As a result, *I. scapularis* ticks are often co-infected with *B. microti* and *B. burgdorferi* s.l.^57^. In New England, USA, the spread of human babesiosis appears to be strongly dependent on Lyme disease^58^. In Europe, co-infections of pathogens in *I. ricinus* ticks also appears to be common^59^. However, co-infections between *B. burgdorferi* s.l. and *A. phagocytophilum* in *I. ricinus* ticks were rare in one study along the west coast of Norway^40^, and this pattern is consistent with what is known about their different reservoir hosts in this region.

Lyme disease incidence will to a large extent reflect the density of nymphal ticks, as the *Borrelia* pathogen has a broad distribution. The more restricted patterns of livestock disease incidence are therefore likely due to either pathogen distribution or land use practices determining the extent to which livestock use areas with infected ticks. The role of exposure in disease emergence has been difficult to assess and is considered the last ‘black hole’ in our knowledge of Lyme disease^60^. Using a comparative approach that included cattle and sheep allowed us to test the role of exposure in contracting tick-borne disease. Sheep are susceptible to anaplasmosis, whereas cattle can acquire both anaplasmosis and babesiosis. The strong spatial correlation between anaplasmosis and babesiosis in cattle suggests a strong role of tick exposure at local scales, which is possibly related to land use practices, rather than tick or pathogen distribution. This interpretation is also supported by the lower correlation of anaplasmosis between sheep and cattle, which should have been high if pathogen distribution was a strong limitation for disease incidence. In our setting, we assume that exposure is mainly related to behavioural aspects of hosts through space use determining the probability of tick encounter (Fig. 1). Space use may be important in determining encounter rates with infected nymphs for both reservoir hosts and dead-end hosts such as humans. For example, male rodents have higher exposure to ticks due to larger home range sizes than female rodents^61^. Staying on wide paths in the forest can reduce human exposure^62^. For livestock, encounter rates is determined by the farmer’s land use practices, which in turn can vary with the landscape. For sheep, exposure is likely limited in many areas where sheep are grazing in the mountains during summer, and hence outside the main distribution of ticks along the west coast of Norway^63,64^. However, sheep are often exposed to ticks during spring when they are released on the infields close to the farm before being sent to the main summer pastures at higher elevations^65^.

Contracting an infection requires encountering an infected tick in the habitat, but also the subsequent successful transfer of the pathogen (Fig. 1). Variation in tick encounter rates between different vertebrate hosts may be caused by differences in host behavior or by ticks having preferences for certain hosts^60,66^. Differences in host grooming behavior, ability to kill ticks, and immune defences among vertebrate hosts may further hinder successful transfer of tick-borne pathogens^67^. The early removal of ticks is certainly important for humans to avoid pathogen transfer^68^. Another important difference between livestock and humans is the number of tick bites per individual, which is probably much higher in livestock. Higher number of tick bites on livestock may explain why the incidence of anaplasmosis in sheep and cattle is much higher than the incidence of Lyme borreliosis in humans, even though the prevalence of *A. phagocytophilum* in ticks is much lower than that of *B. burgdorferi* s.l.

The finding that exposure plays a large role in livestock disease raises concerns given the development of new practices for the more ethical stocking of livestock. Over the last 5 years, the number of cattle grazing on outfields has increased by 8.7% in Norway (“Statens landbruksforvaltning”), and as of January 1, 2014, it is mandatory to pasture cattle for a minimum of eight weeks. A more environmentally friendly way of producing livestock may come at a cost of an increased incidence of tick-borne diseases. In contrast, more intense production will lead to more dairy cattle grazing on cultivating pasture that does not have forest vegetation, which will reduce contact with ticks. Since 2002, when the milk quota was put up for sale in Norway, there have been increases in herd size, in dairy production and in milk yield. Furthermore, since 2005, there has been a large increase in the use of automatic milking systems (AMS). The decreasing trend in babesiosis and anaplasmosis in cattle from 2005 onwards suggests reduced tick exposure due to more intensive production (Fig. 2).

The same main pattern of emergence across the tick-borne diseases suggests some shared underlying limiting factor and that uncertainty in the data did not cause severe bias. Nevertheless, caution regarding observation processes is necessary when comparing different disease records. Processes that potentially create bias may involve variation in 1) diagnostic criteria, 2) reporting and/or 3) treatment; in the present study, we considered these potential sources of bias in detail for each disease (cfr. Supplementary Discussion). It is more difficult to diagnose anaplasmosis in live animals, due to weaker clinical symptoms, than it is to diagnose Lyme disease and babesiosis. The very similar patterns of emergence and drivers of anaplasmosis and babesiosis in cattle nevertheless suggest that these data are of parallel reliability over the duration of the study. In addition, the number of cases of babesiosis and anaplasmosis declined over time, despite improvements to the recording system over the duration of the study. These potential sources of uncertainty should not affect the spatial patterns. The main patterns reported in this study are therefore unlikely to have been biased by observation error.

Our study reveals relationships among livestock and human diseases and is consistent with a One Health perspective^4^. As shown in our study, a consistent way to quantify and determine the drivers of emerging infectious diseases has implications for the development of disease mitigation and prevention strategies^69^. For example, reducing deer density is possible, and targeting deer might also remove reservoir hosts of *A. phagocytophilum*. In contrast, it will be more difficult to fight climate change and to reduce the positive effects of global warming on the distribution and abundance of the common tick vector. The warming climate combined with a new green environmental policy requiring more outfield grazing presents a challenge. However, the reductions in both anaplasmosis and babesiosis in the last few years suggest that farmers are able to at least partly mitigate the diseases. The role of exposure in the pattern of disease incidence in livestock suggests that land use practices and landscape management strategies will be important mitigation tools^70^.

## Methods

### Study area

The tick-borne disease data span the whole of Norway and thus represent a variety of climates and ecosystems, covering a latitudinal range of 57°58′–71°08′N. For a detailed description of the geology, climate and host populations, we refer to a recent paper^37^. In brief, the climate is colder further north, whereas the mountain range in the south separates the western coastal region from the inland (eastern) region, which has a more continental climate (drier and colder). The vegetation includes smaller areas of nemoral forest along the southernmost coast, e.g., deciduous forest trees such as oak (*Quercus* spp.) that require warmer habitat, whereas further inland and towards the east, the forest is boreonemoral with boreal zones.

### Lyme disease in humans

Human cases of tick-borne Lyme disease (borreliosis) were retrieved from the Norwegian Surveillance System for Communicable Diseases (MSIS) for the time period 1991 to 2015 (n = 3424 cases). We limited the data to cases for which the municipality of tick-bite was confirmed (n = 2057). In Norway, it is compulsory to report laboratory-confirmed cases of Lyme disease to the Norwegian Institute of Public Health (NIPH)^34^. The definition of a confirmed case has changed over time. We restricted the data to 1995 onwards; during this period, only disseminated disease/chronic manifestations of Lyme disease were notifiable^34^. The compiled data was previously used to assess temporal changes in the distribution of ticks^34^ and the role of deer populations in the emergence of Lyme disease^37^.

### Anaplasmosis and babesiosis in cattle

Records of the tick-borne diseases bovine babesiosis (n = 2436) and anaplasmosis (n = 1616) are available from the Norwegian Cattle Health Recording system (NCHRS) for the time period 1996 to 2015^71^. This system is considered to be highly reliable. The records only include animals that were treated by veterinarians. The data on babesiosis were used previously to assess distribution changes in the tick *I. ricinus* in Norway^34^.

### Anaplasmosis in sheep

Cases of the tick-borne disease anaplasmosis were retrieved from the Sheep Recording System database (“Sauekontrollen”) in Norway (n = 1222). Data on sheep body mass and litter size from this database have been widely used in ecological research^72–75^, but the data on anaplasmosis have not been analysed. Similar to the NCHRS, this database includes only records in which the animals were treated by veterinarians and reported by the sheep farmer. We removed 5 records of obvious miscoding (set as >70 cases of anaplasmosis in the same herd in a given year), as the entire herd of one farmer was notified to have anaplasmosis in one year, or even two subsequent years and not in other years. These records were likely miscoded for the treatment of ectoparasites. For the retained data, we included a covariate to control for the general increase in reporting of diseases over time and thus avoid bias.

### Calculating incidence

To understand the emergence of tick-borne diseases, both the number of cases and the incidence are of interest. The number of cases is clearly linked to the population sizes of humans, cattle and sheep (which are needed to calculate incidence) as well as exposure. (1) The numbers of people were retrieved from Statistics Norway. In a few cases, numbers were lacking due to the merging of municipalities; in these cases, the estimate closest in time was used. (2) The numbers of cattle in total and in grazing outfields per municipality and year were obtained from Statistics Norway (1990–1999) and Landbruksdirektoratet (2000–2015). The data are derived from applications for a production subsidy, which is submitted each year on July 31. The livestock industry is subsidized in Norway, and as this subsidy is an important contributor to a farmer’s income, the data are regarded as highly reliable. Due to privacy issues, the exact numbers of each livestock category are not reported if there is only single user within a municipality. However, these are identifiable cases, and we imputed 10 outfield-grazing cattle. This imputation was performed only for 1–4 years and for 3 municipalities with cases of anaplasmosis or babesiosis. (3) The number of sheep grazing in the outfields was available at the municipality scale for each year from 1999 to 2015 from Statistics Norway. However, since not all of the sheep were represented in the Sheep Control database from which we extracted the anaplasmosis data, when calculating the incidence of anaplasmosis we only included those sheep represented in the Sheep Control database.

### Spatial and habitat covariates

Tick density is strongly related to distance from the coast, latitude and altitude in Norway^34,49,64^. We therefore retrieved data on latitude and distance from the fjord for each municipality^37^. We used Universal Transverse Mercator (UTM) coordinates to measure latitude. Ticks are abundant up to approximately 200–250 m above sea level; therefore, we produced metrics on the proportion of area below 200 m for each municipality. In addition, we retrieved data on the proportions of forested area, agricultural land and human settlement in each municipality from Statistics Norway.

### Deer density

Data on the numbers of harvested moose (*Alces alces*), red deer and roe deer at the scale of municipalities are available from Statistics Norway. In Norway, the management strategy for cervids includes the aim of an approximately proportional harvest relative to population size. There is thus good evidence that the harvest number is strongly correlated with the overall cervid population size and that it does not merely reflect the size of the harvest quota for roe deer^76,77^, red deer^78^ and moose^79^. To obtain a measure of deer population density, we divided the number of deer by the area of deer habitat defined by the management strategy, which provides the basis for the harvest quotas (termed qualifying area). This index has been widely used in demographic studies of deer in Norway^72,80^. We used the same data as in Mysterud *et al*.^37^. When testing for a spatial deer density component, we used the mean deer density of the municipality over the whole time period for either the whole cervid community or for red deer and roe deer specifically. For Lyme disease, there was sufficient data to separate the spatial and temporal components of deer density^37^. We defined the temporal component of deer density as the residual from the mean deer density in a municipality a given year^37^.

### Climate covariates

We retrieved the broad climate covariate, the North Atlantic Oscillation (NAO), which is well known to affect the climate in Norway. We used the principal component analysis-based data from Jim Hurrell at the National Center for Atmospheric Research (https://climatedataguide.ucar.edu/climate-data/hurrell-north-atlanticoscillation-nao-index-pc-based) on seasonal NAO for December–January–February, March–April–May, June–July–August and September–October–November. We considered time lags of up to 2 years to account for the life cycle of ticks, where events that influence the population density of reservoir hosts will influence the density of infected nymphs two years later^81^.

### Statistical analyses

Data were analysed using the R × 64 v. 3.1.2 software (http://www.r-project.org/). Initially, regional patterns of disease incidence over time were described by fitting smoothing splines by Generalized Additive Models (GAM) with a quasi-Poisson error distribution using the R package mgcv^82^. There was no case of human Lyme disease recorded in 46.5% of all 402 municipalities in Norway (excluding 26 municipalities with no sheep or cattle in the livestock databases); this percentage was 82.1% for anaplasmosis in sheep, 68.7% for anaplasmosis in cattle, and 64.4% for babesiosis. Due to the spatially restricted distributions of the tick-borne diseases, we modelled both disease occurrence and disease incidence. We analysed disease occurrence with logistic mixed-effects regressions using the R package lme4. We excluded 9 (of 19) counties because they had fewer than 10 cases for each of the tick-borne diseases of livestock. Mixed effects were either municipality (for anaplasmosis in cattle and sheep) or municipality nested in county (for Lyme disease and babesiosis). Disease incidence data were modelled with negative binomial mixed effects regressions using the R package glmmADMB^83^ and were restricted to 192 (of 253) municipalities with at least 1 case of tick-borne disease. Thus, our response variable was the number of cases of known location of infection. For Lyme disease, we restricted the analysis to those cases in which the patient reported the municipality of the tick bite. For sheep, location of infection is known based on data on the area for outfield grazing. For cattle, the farm is the location of the herd. This approach is unlikely to yield bias because it is very rare for farmers to move their cattle over long distances from the farm for grazing. We used as an offset the (natural log-transformed) number of people, sheep or cattle (population) in each municipality, so that we were in effect modelling incidence. Municipality, or municipalities nested within county, were included as random intercepts.

To quantify trends, we added “year” as a continuous variable; we also tried “year” as a second order term. We included quite a wide range of environmental covariates and used the Akaike Information Criterion (AIC) in model selection to select the most parsimonious model. We present in the Supplementary Tables S1-S4 how excluding parameters from the final, best models affected the ΔAIC for each tick-borne disease. Collinearity was assessed by calculating variance inflation factors (VIF’s^84^), and only variables having VIFs < 4 were retained in a given model.

Model fit was evaluated by plotting the residuals against the predicted values and by plotting the residuals of the final models of incidence against each of the explanatory variables. To achieve a good fit, several explanatory variables were natural log-transformed or square root-transformed to linearize their relationships with the response variable. We checked for autocorrelation structure in both space (using previous year’s presence/absence of disease in neighbouring municipalities) and time (incidence lagged by one year).

Pearson correlation with bootstrap confidence intervals were used to quantify 1) spatial correlations of total disease cases and mean incidence over time (n = 402 municipalities) and 2) temporal correlations between first-differenced series for all of Norway, i.e., the change in total number of cases or incidence for all of Norway from one year to the next. Lastly, 3) region-wise synchrony was quantified by calculating the mean correlation among model residuals from 60 municipalities showing at least one Lyme disease case and one livestock-disease case (1996–2015), by using the R package ‘ncf’^85^. The model residuals were from mixed effects models with negative binomial errors that controlled for the spatial variables and the main temporal trend (Table 2A).

### Data availability

LD incidence data were derived from the Norwegian Surveillance System for Communicable Diseases (MSIS) and are available from the Norwegian Institute of Public Health (http://www.fhi.no/artikler/?id=93861). Data on human demography, land use, and host populations are available from Statistics Norway (https://www.ssb.no/statistikkbanken). Data on anaplasmosis in sheep is available upon request from Animalia. Data on anaplasmosis and babesiosis were obtained from the Norwegian Dairy Herd Recording System, TINE SA.

## Electronic supplementary material

Supplementary info

## References

[1] Taylor LH, Latham SM, Woolhouse MEJ (2001). Risk factors for human disease emergence. Phil Trans R Soc London Ser B.

[2] Jones KE, Patel NG, Levy MA (2008). Global trends in emerging infectious diseases. Nature.

[3] Gortazar C, Reperant LA, Kuiken T (2014). Crossing the interspecies barrier: opening the door to zoonotic pathogens. Plos Pathog.

[4] Atlas, R. M. & Maloy, S. O*ne health. People, animals, and the environment*. (American Society forMicrobiology, Washington DC, 2014).

[5] Radolf JD, Caimano MJ, Stevenson B, Hu LT (2012). Of ticks, mice and men: understanding the dual-host lifestyle of Lyme disease spirochaetes. Nature Rev Microbiol.

[6] Piesman J, Gern L (2004). Lyme borreliosis in Europe and North America. Parasitology.

[7] Ruiz-Fons F, Fernandez-de-Mera IG, Acevedo P, Gortázar C, de la Fuente J (2012). Factors driving the abundances of Ix*odes ricinus t*icks and the prevalence of Zoonotic I. *ricinus-b*orne pathogens in natural foci. Appl Environ Microbiol.

[8] Medlock JM, Hansford KM, Bormane A (2013). Driving forces for changes in geographical distribution of Ix*odes ricinus t*icks in Europe. Parasite Vector.

[9] Werden L, Barker IK, Bowman J (2014). Geography, deer, and host biodiversity shape the pattern of Lyme disease emergence in the Thousand Islands Archipelago of Ontario, Canada. Plos One.

[10] Dobson ADM, Randolph SE (2011). Modelling the effects of recent changes in climate, host density and acaricide treatments on population dynamics of I*xodes ricinus i*n the UK. J Appl Ecol.

[11] Wang H-H, Grant WE, Teel PD (2012). Simulation of climate-host-parasite-landscape interactions: a spatially explicit model for ticks (Acari: Ixodidae). Ecol Mod.

[12] Olwoch JM, Reyers B, van Jaarsveld AS (2009). Host-parasite distribution patterns under simulated climate: implications for tick-borne diseases. Int J Clim.

[13] Ostfeld RS, Keesing F (2012). Effects of host diversity on infectious disease. Ann Rev Ecol Evol Syst.

[14] Wood CL, Lafferty KD (2013). Biodiversity and disease: a synthesis of ecological perspectives on Lyme disease transmission. Trends Ecol Evol.

[15] Wood CL (2014). Does biodiversity protect humans against infectious disease?. Ecol.

[16] Ostfeld RS, Keesing F (2013). Straw men don’t get Lyme disease: response to Wood and Lafferty. Trends Ecol Evol.

[17] Randolph SE (2013). Commentary on ‘A candide response to Panglossian accusations by Randolph and Dobson: biodiversity buffers disease’ by Dr. R. Ostfeld (*Parasitology* 2013, in press). Parasitology.

[18] Mannelli A, Bertolotti L, Gern L, Gray J (2012). Ecology of Bo*rrelia burgdorferi se*nsu lato in Europe: transmission dynamics in multi-host systems, influence of molecular processes and effects of climate change. FEMS Microbiol Rev.

[19] Ostfeld RS (2014). Life history and demographic drivers of reservoir competence for three tick-borne zoonotic pathogens. Plos One.

[20] Woldehiwet Z (2010). The natural history of An*aplasma phagocytophilum*. Vet Parasitol.

[21] Zintl A, Mulcahy G, Skerrett HE, Taylor SM, Gray JS (2003). Ba*besia divergens*, a bovine blood parasite of veterinary and zoonotic importance. Clin Microbiol Rev.

[22] Blanco JR, Oteo JA (2002). Human granulocytic ehrlichiosis inEurope. Clin Microbiol Infec.

[23] Ostfeld RS, Brunner JL (2015). Climate change and Ix*odes ti*ck-borne diseases of humans. Phil Trans R Soc London Ser B.

[24] van Duijvendijk G, Sprong H, Takken W (2015). Multi-trophic interactions driving the transmission cycle of Bo*rrelia afzelii b*etween Ix*odes ricinus and rodents: a review*. Parasites & Vectors.

[25] Tälleklint L, Jaenson TGT (1994). Transmission of Bo*rrelia burgdorferi s*.l. from mammal reservoirs to the primary vector of Lyme borreliosis, Ix*odes ricinus (*Acari, Ixodidae), in Sweden. J Med Entomol.

[26] Strandh M, Råberg L (2015). Within-host competition between Bo*rrelia afzelii ospC s*trains in wild hosts as revealed by massively parallel amplicon sequencing. Phil Trans R Soc London Ser B.

[27] Råberg L, Hagström Å, Andersson M (2017). Evolution of antigenic diversity in the tick-transmitted bacterium Bo*rrelia afzelii:* a role for host specialization?. J Evol Biol.

[28] Stuen S, Granquist EG, Silaghi C (2013). An*aplasma phagocytophilum -* a widespread multi-host pathogen with highly adaptive strategies. Front Cell Infect Microbiol.

[29] Scharf W, Schauer S, Freyburger F (2011). Distinct host species correlate with An*aplasma phagocytophilum a*nkA gene clusters. J Clin Microbiol.

[30] Jahfari S, Coipan EC, Fonville M (2014). Circulation of four An*aplasma phagocytophilum e*cotypes in Europe. Parasite Vector.

[31] Dugat T, Chastagner A, Lagrée A-C (2014). A new multiple-locus variable-number tandem repeat analysis reveals different clusters for An*aplasma phagocytophilum c*irculating in domestic and wild ruminants. Parasite Vector.

[32] Duh D, Petrovec M, Bidovec A, Avsic-Zupanc T (2005). Cervids as Ba*besia ho*sts, Slovenia. Emerg Infect Dis.

[33] Funk S, Bogich TL, Jones KE, Kilpatrick AM, Daszak P (2013). Quantifying trends in disease impact to produce a consistent and reproducible definition of an emerging infectious disease. Plos One.

[34] Jore S, Viljugrein H, Hofshagen M (2011). Multi-source analysis reveals latitudinal and altitudinal shifts in range of Ix*odes ricinus a*t its northern distribution limit. Parasite Vector.

[35] Karlsen SR, Høgda K-A, Wielgolaski F-E (2009). Growing-season trends in Fennoscandia 1982-2006, determined from satellite and phenology data. Climate Research.

[36] Li S, Gilbert L, Harrison PA, Rounsevell MDA (2016). Modelling the seasonality of Lyme disease risk and the potential impacts of a warming climate within the heterogeneous landscapes of Scotland. J Roy Soc Interface.

[37] Mysterud A (2016). Contrasting emergence of Lyme disease across ecosystems. Nature Comm.

[38] Gern L, Estrada-Peña A, Frandsen F (1998). European reservoir hosts of Bo*rrelia burgdorferi s*ensu lato. Zentralblatt für Bakteriologie.

[39] Kjelland V, Stuen S, Skarpaas T, Slettan A (2010). Prevalence and genotypes of Bo*rrelia burgdorferi s*ensu lato infection in Ix*odes ricinus t*icks in southern Norway. Scand J Infect Dis.

[40] Mysterud A, Easterday WR, Qviller L, Viljugrein H, Ytrehus B (2013). Spatial and seasonal variation in prevalence of An*aplasma phagocytophilum a*nd Bo*rrelia burgdorferi in* Ix*odes ricinus* ticks in Norway. Parasite Vector.

[41] Hvidsten D, Stuen S, Jenkins A (2014). Ix*odes ricinus a*nd Bo*rrelia pr*evalence at the Arctic Circle in Norway. Ticks Tick Borne Dis.

[42] Schorn S, Pfister K, Reulen H (2011). Prevalence of An*aplasma phagocytophilum i*n I*xodes ricinus i*n Bavarian public parks, Germany. Ticks Tick Borne Dis.

[43] Lempereur L, Lebrun M, Cuvelier P (2012). Longitudinal field study on bovine Ba*besia sp*p. and An*aplasma phagocytophilum i*nfections during a grazing season in Belgium. Parasitol Res.

[44] Henningsson A (2015). Detection of An*aplasma phagocytophilum i*n I*xodes ricinus t*icks from Norway using a realtime PCR assay targeting the An*aplasma ci*trate synthase gene gltA. BMC Microbiology.

[45] Seland, I. V. The distribution of ticks and tick-borne pathogens from coast to inland in southeast Norway. (Master thesis, University of Oslo., Oslo, 2016).

[46] Rosef O, Radzijevskaja J, Paulauskas A, Haslekås C (2009). The prevalence of An*aplasma phagocytophilum i*n host-seeking Ix*odes ricinus t*icks in Norway. Clin Microbiol Infec.

[47] Soleng A, Kjelland V (2013). Bo*rrelia burgdorferi s*ensu lato and An*aplasma phagocytophilum i*n I*xodes ricinus t*icks in Brønnøysund in northern Norway. Ticks Tick Borne Dis.

[48] Stuen S (2013). An*aplasma phagocytophilum v*ariants in sympatric red deer (Ce*rvus elaphus)* and sheep in southern Norway. Ticks Tick Borne Dis.

[49] Jore S, Vanwambeke SO, Viljugrein H (2014). Climate and environmental change drives Ix*odes ricinus g*eographical expansion at the northern range margin. Parasite Vector.

[50] Chastagner A, Pion A, Verheyden H (2017). Host specificity, pathogen exposure, and superinfections impact the distribution of An*aplasma phagocytophilum g*enotypes in ticks, roe deer, and livestock in a fragmented agricultural landscape. Infection, Genetics and Evolution.

[51] Andersson, M. O. *et al*. Co-infection with Ba*besia divergens a*nd An*aplasma phagocytophilum i*n cattle (Bo*s taurus)*, Sweden. Ti*cks Tick Borne Dis i*n press, (2017). 10.1016/j.ttbdis.2017.08.005 28869191

[52] Øines Ø, Radzijevskaja J, Paulauskas A, Rosef O (2012). Prevalence and diversity of Ba*besia sp*p. in questing Ix*odes ricinus t*icks from Norway. Parasite Vector.

[53] Yabsley MJ, Shock BC (2013). Natural history of zoonotic Ba*besia:* role of wildlife reservoirs. In*ternational*. Journal of Parasitology: Parasites and Wildlife.

[54] Hasle G (2010). Detection of Ba*besia divergens i*n southern Norway by using an immunofluorescence antibody test in cow sera. Acta Vet Scand.

[55] Cézanne R (2016). Molecular analysis of Ana*plasma phagocytophilum an*d Bab*esia divergens in* red deer (Cer*vus elaphus) i*n Western Austria. Molecular and Cellular Probes.

[56] Malandrin L, Jouglin M, Sun Y, Brisseau N, Chauvin A (2010). Redescription of Ba*besia capreoli (*Enigk and Friedhoff, 1962) from roe deer (Ca*preolus capreolus):* Isolation, cultivation, host specificity, molecular characterisation and differentiation from Ba*besia divergens*. International Journal for Parasitology.

[57] Hersh MH, Ostfeld RS, McHenry DJ (2014). Co-infection of blacklegged ticks with Ba*besia microti a*nd Bo*rrelia burgdorferi is* higher than expected and acquired from small mammal hosts. Plos One.

[58] Walter, K. S., Pepin, K. M., Webb, C. T. *et al*. Invasion of two tick-borne diseases across New England: harnessing human surveillance data to capture underlying ecological invasion processes. Pr*oceedings of the Royal Society of London B: Biological Sciences* **283** (2016). 10.1098/rspb.2016.0834 PMC4920326 27252022

[59] Diuk-Wasser MA, Vannier E, Krause PJ (2016). Coinfection by Ixodes tick-borne pathogens: Ecological, epidemiological, and clinical consequences. Trends Parasitol.

[60] Parham PE, Waldock J, Christophides GK (2015). Climate, environmental and socio-economic change: weighing up the balance in vector-borne disease transmission. Phil Trans R Soc London Ser B.

[61] Perkins SE, Cattadori IM, Tagliapietra V, Rizzoli AP, Hudson PJ (2003). Empirical evidence for key hosts in persistence of a tick-borne disease. Int J Para.

[62] Walker AR, Alberdi MP, Urquhart KA, Rose H (2001). Risk factors in habitats of the tick Ix*odes ricinus i*nfluencing human exposure to Eh*rlichia phagocytophila b*acteria. Med Vet Entomol.

[63] Qviller L, Grøva L, Viljugrein H, Klingen I, Mysterud A (2014). Temporal pattern of questing tick Ix*odes ricinus d*ensity at differing elevations in the coastal region of western Norway. Parasite Vector.

[64] Qviller L, Risnes-Olsen N, Bærum KM (2013). Landscape level variation in tick abundance relative to seasonal migration pattern of red deer. Plos One.

[65] Gilbert L, Brunker K, Lande U, Klingen I, Grøva L (2017). Environmental risk factors for Ixodes ricinus ticks and their infestation on lambs in a changing ecosystem: Implications for tick control and the impact of woodland encroachment on tick-borne disease in livestock. Agriculture, Ecosystems & Environment.

[66] McCoy KD, Leger E, Dietrich M (2013). Host specialization in ticks and transmission of tick-borne diseases: a review. Front Cell Infect Microbiol.

[67] Keesing F, Brunner J, Duerr S (2009). Hosts as ecological traps for the vector of Lyme disease. Proc R Soc Lond Ser B.

[68] Zöldi V, Turunen T, Lyytikäinen O, Sane J (2017). Knowledge, attitudes, and practices regarding ticks and tick-borne diseases, Finland. Ticks Tick Borne Dis.

[69] Rosenthal SR, Ostfeld RS, McGarvey ST, Lurie MN, Smith KF (2015). Redefining disease emergence to improve prioritization and macro-ecological analyses. One Health.

[70] Lambin EF, Tran A, Vanwambeke SO, Linard C, Soti V (2010). Pathogen landscapes: interactions between land, people, disease vectors, and their animal hosts. International Journal of Health Geographics.

[71] Østerås O (2007). Results and evaluation of thirty years of health recordings in the Norwegian dairy cattle population. J Dairy Sci.

[72] Mysterud A, Stenseth NC, Yoccoz NG, Langvatn R, Steinheim G (2001). Nonlinear effects of large-scale climatic variability on wild and domestic herbivores. Nature.

[73] Mysterud A, Steinheim G, Yoccoz NG, Holand Ø, Stenseth NC (2002). Early onset of reproductive senescence in domestic sheep (Ov*is aries)*. Oikos.

[74] Nielsen A, Yoccoz NG, Steinheim G (2012). Are responses of herbivores to environmental variability spatially consistent in alpine ecosystems?. Global Change Biol.

[75] Steinheim G, Mysterud A, Holand Ø, Bakken M, Ådnøy T (2002). The effect of initial weight of the ewe on later reproductive effort in domestic sheep (Ov*is aries)*. J Zool.

[76] Grøtan V, Sæther B-E, Engen S (2005). Climate causes large-scale spatial synchrony in population fluctuations of a temperate herbivore. Ecol.

[77] Mysterud A, Østbye E (2006). The effect of climate and density on individual and population growth of roe deer Ca*preolus capreolus a*t northern latitudes - the Lier valley, Norway. Wildl Biol.

[78] Mysterud A, Meisingset EL, Veiberg V (2007). Monitoring population size of red deer: an evaluation of two types of census data from Norway. Wildl Biol.

[79] Solberg EJ, Sæther B-E, Strand O, Loison A (1999). Dynamics of a harvested moose population in a variable environment. J Anim Ecol.

[80] Mysterud A, Yoccoz NG, Langvatn R, Pettorelli N, Stenseth NC (2008). Hierarchical path analysis of deer responses to direct and indirect effects of climate in northern forest. Phil Trans R Soc London.

[81] Ostfeld RS, Canham CD, Oggenfuss K, Winchcombe RJ, Keesing F (2006). Climate, deer, rodents, and acorns as determinants of variation in Lyme-disease risk. Plos Biol.

[82] Wood, S. *Generalized additive models: an introduction with R*. (Chapman & Hall, Boca Raton, 2006).

[83] Skaug, H. Fournier, D. & Nielsen, A. gl*mmADMB: Generalized linear mixed models using AD Model Builder*. (http://glmmadmb.r-forge.r-project.org/, 2006).

[84] James, G. Witten, D. Hastie, T. & Tibshirani, R. *An introduction to statistical learning with applications in R*. (Springer, New York, 2013).

[85] Bjørnstad, O. N. Package ‘ncf’. Spatial nonparametric covariance functions. (2016).

