# Supplementary material for: Emergence of tick-borne diseases at northern latitudes in Europe: a comparative approach
Source: Sci Rep. 2017 Nov 24;7:16316. doi: 10.1038/s41598-017-15742-6 (PMC5701145; doi:10.1038/s41598-017-15742-6)
Supplement: Supplementary file 1 — Supplementary info [file 41598_2017_15742_MOESM1_ESM.pdf]

# Emergence of tick-borne diseases at northern latitudes in Europe: a comparative approach

Atle Mysterud, Solveig Jore, Olav Østerås, Hildegunn Viljugrein

## Supplementary information

Content:

- Supplementary Figures 1–3
- Supplementary Tables 1–4
- Supplementary Discussion
- Supplementary References

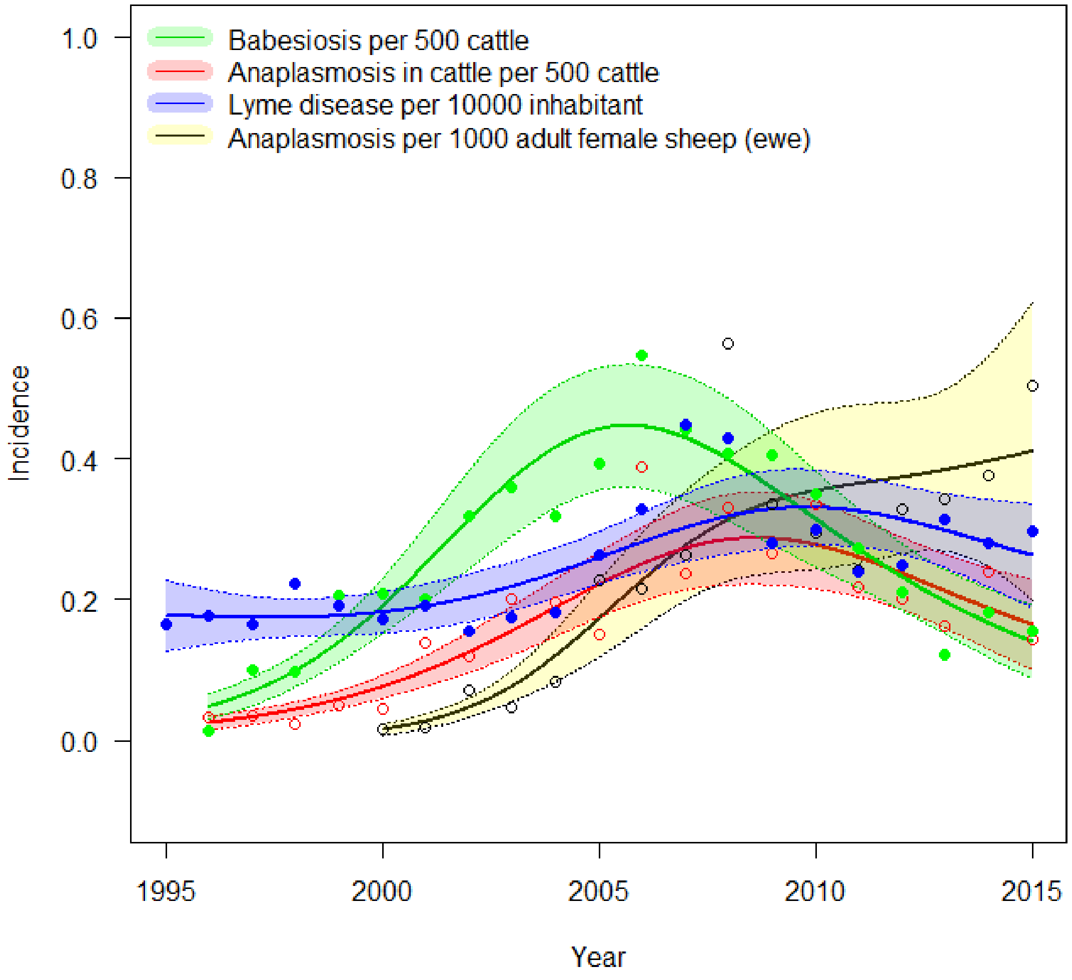


**Supplementary Figure 1| Pattern of disease incidence over time for Lyme disease in humans, live-stock fever in sheep and anaplasmosis and babesiosis in cattle analysed at the national scale in Norway, which is at a broader spatial scale than the municipality scale presented in the main analysis (in figure 2).**

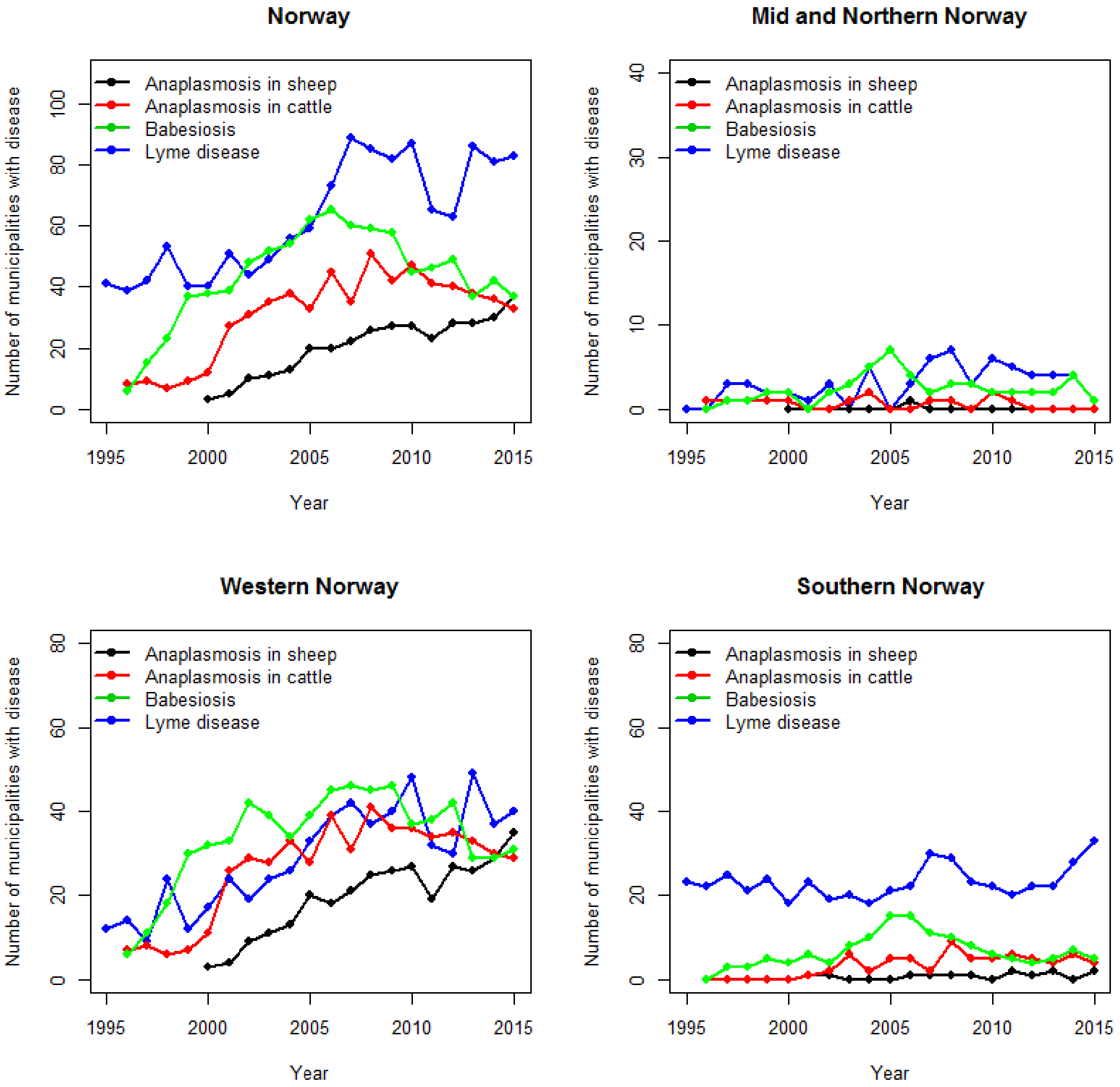


**Supplementary Figure 2 | The number of municipalities with disease cases.** These are raw data for both the whole of Norway, as well as for each region of Norway from 1995-2015.

**Supplementary Figure 3 | Mean number of human inhabitants, sheep and cattle, and mean cervid, roe deer and red deer density in each municipality of Norway 1995-2005.**

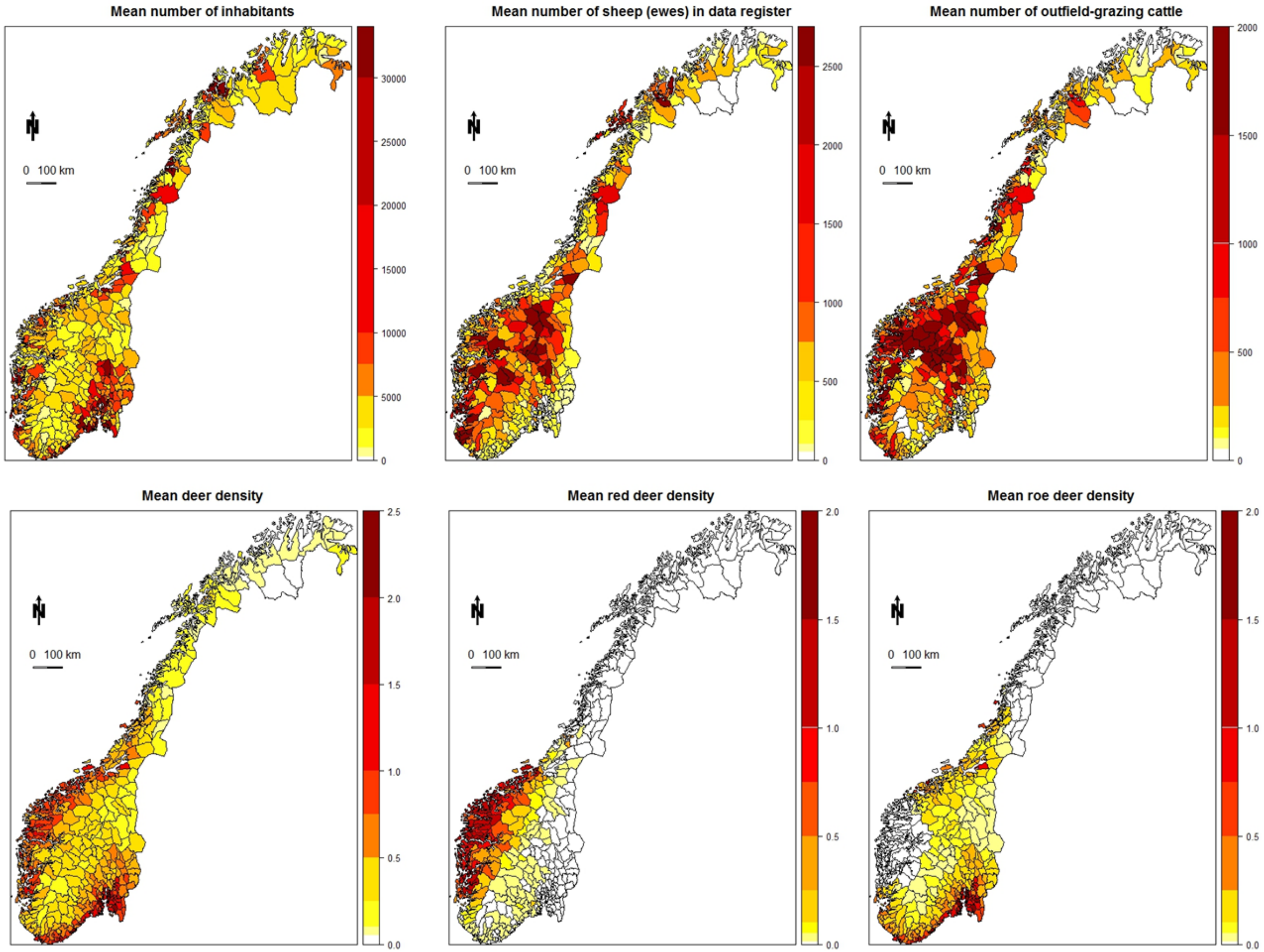

**Supplementary Table 1 | Model of Lyme disease in humans.** Parameter estimates of incidence from mixed-effects model using (A) counts in a negative binomial model and (B) presence or absence of disease in a logistic regression model of Lyme disease in humans from 10 counties in Norway from years 1995-2015.

| Parameter | Estimate | SE | z | p | ΔAIC |
|---|---|---|---|---|---|
| A. Negative binomial model | | | | | |
| Intercept | -10.1714 | 0.1547 | -65.753 | <0.001 | |
| year | 0.4308 | 0.0530 | 8.134 | <0.001 | 78.3 |
| $year^2$ | -0.2052 | 0.0438 | -4.680 | <0.001 | 21.1 |
| Region «South» | 0.2385 | 0.3529 | 0.676 | 0.499 | 25.9 |
| sqrt(temporal autocorrelation) | 0.0642 | 0.0278 | 2.311 | 0.021 | 3.3 |
| sqrt(prop(agriculture)) | -0.3148 | 0.0767 | -4.106 | <0.001 | 14.3 |
| sqrt(prop(human settlement)) | -0.4597 | 0.0581 | -7.909 | <0.001 | 42.5 |
| NAO-DJF (lag 1 yr) | 0.0720 | 0.0230 | 3.133 | 0.002 | 8.0 |
| NAO-MAM (lag 2 yr) | -0.0771 | 0.0231 | -3.333 | 0.001 | 9.1 |
| log(mean spatial deer density + 0.01) | 0.4412 | 0.0748 | 5.895 | <0.001 | 34.1 |
| temporal deer density | 0.1081 | 0.0421 | 2.569 | 0.010 | 4.7 |
| North-UTM | -0.5933 | 0.1425 | -4.163 | <0.001 | 23.0 |
| prop(area >200 m a.s.l.) | -0.3660 | 0.0954 | -3.838 | <0.001 | 11.1 |
| sqrt(distance to fjord) | -0.3221 | 0.0935 | -3.446 | 0.001 | 10.3 |
| Region «South» * year | -0.3811 | 0.0697 | -5.468 | <0.001 | 26.9 |
| Region «South» * $year^2$ | 0.1440 | 0.0607 | 2.373 | 0.018 | |
| B. Logistic regression model | | | | | |
| Intercept | -2.4538 | 0.2006 | -12.233 | <0.001 | |
| year | 0.3972 | 0.0475 | 8.364 | <0.001 | 114.5 |
| log(mean no. of humans) | 0.8197 | 0.0750 | 10.932 | <0.001 | 111.5 |
| sqrt(prop(agriculture)) | -0.3095 | 0.0889 | -3.4792 | <0.001 | 9.7 |
| log(mean spatial deer density + 0.01) | 0.6155 | 0.1258 | 4.891 | <0.001 | 20.3 |
| sqrt(distance to fjord) | -0.9600 | 0.1203 | -7.983 | <0.001 | 60.6 |
| North-UTM | -1.4989 | 0.2088 | -7.179 | <0.001 | 27.8 |
| year * log(mean no. humans) | 0.1791 | 0.0489 | 3.659 | <0.001 | 11.5 |

sqrt = square-root transformed; prop = proportion; temporal autocorrelation = incidence of Lyme disease previous year. Continuous variables are scaled to variance=1 and mean=0. NAO = North Atlantic Oscillation index. DJF = December-January-February. MAM = March-April-May. ΔAIC = the increase in AIC if removing the variable. The change in AIC if removing the random intercepts is 111.1 (A) and 154.2 (B). If keeping only the year trend (and for B, mean number of inhabitants), ΔAIC is 134.6 (A) and 148.0 (B).

**Supplementary Table 2 | Model of babesiosis in cattle.** Parameter estimates of incidence from mixed-effects model using (A) counts in a negative binomial model and (B) presence or absence of disease in a logistic regression model of babesiosis in cattle from 10 counties in Norway from years 1996-2015.

| Parameter | Estimate | SE | z | p | ΔAIC |
|---|---|---|---|---|---|
| A. Negative binomial model | | | | | |
| Intercept | -7.9750 | 0.4833 | -16.46 | <0.001 | |
| year | 0.2828 | 0.0400 | 7.07 | <0.001 | 187.8 |
| $year^2$ | -0.5785 | 0.0430 | -13.45 | <0.001 | 181.6 |
| sqrt(temporal autocorrelation) | 0.1979 | 0.0320 | 6.18 | <0.001 | 36.1 |
| NAO-MAM (lag 1 yr) | -0.0719 | 0.0300 | -2.40 | 0.016 | 3.7 |
| log(mean spatial deer density + 0.01) | 0.8820 | 0.1350 | 6.53 | <0.001 | 40.1 |
| sqrt(prop(human settlement)) | -0.4865 | 0.1189 | -4.09 | <0.001 | 15.2 |
| B. Logistic regression model | | | | | |
| Intercept | -4.0443 | 0.4304 | -9.40 | <0.001 | |
| year | 0.2534 | 0.0534 | 4.75 | <0.001 | 20.8 |
| temporal autocorrelation > 0 | 1.0131 | 0.1206 | 8.40 | <0.001 | 67.8 |
| log(mean no. of cattle + 1) | 1.1487 | 0.1272 | 9.03 | <0.001 | 85.8 |
| log(mean spatial deer density + 0.01) | 0.7088 | 0.1708 | 4.15 | <0.001 | 15 |
| North-UTM | -1.0502 | 0.3503 | -3.00 | 0.003 | 4.1 |
| sqrt(distance to fjord) | -0.3641 | 0.1786 | -2.04 | 0.04 | 2.2 |
| Region “West” | 1.2748 | 0.6433 | 1.98 | 0.05 | 1.6 |

sqrt = square-root transformed; temporal autocorrelation = incidence of babesiosis previous year. Continuous variables are scaled to variance=1 and mean=0. NAO = North Atlantic Oscillation index. MAM = March-April-May. ΔAIC = the increase in AIC if removing the variable. Random effects are given by 192 (A) and 253 (B) municipalities nested in 10 counties. The change in AIC if removing the random intercepts is 605.9 (A) and 583.2 (B). If keeping only the year trend (and mean no. of cattle in B), ΔAIC is 99.6 (A) and 109.4 (B).

**Supplementary Table 3 | Model of anaplasmosis in cattle.** Parameter estimates of incidence from mixed-effects model using (A) counts in a negative binomial model and (B) presence or absence of disease in a logistic regression model of anaplasmosis in cattle from 10 counties in Norway from years 1996-2015. ΔAIC = the increase in AIC if removing the variable.

| Parameter | Estimate | SE | z | p | ΔAIC |
|---|---|---|---|---|---|
| A. Negative binomial model | | | | | |
| Intercept | -8.7486 | 0.4840 | -18.08 | <0.001 | |
| year | 0.6735 | 0.0589 | 11.44 | <0.001 | 166.5 |
| $year^2$ | -0.5047 | 0.0529 | -9.53 | <0.001 | 91.9 |
| sqrt(temporal autocorrelation) | 0.1288 | 0.0366 | 3.52 | <0.001 | 10.5 |
| NAO-DJF (lag 1 yr) | 0.0720 | 0.0361 | 2.00 | 0.046 | 2.0 |
| log(mean spatial deer density + 0.01) | 0.5715 | 0.1365 | 4.19 | <0.001 | 15.1 |
| B. Logistic regression model | | | | | |
| Intercept | -4.7984 | 0.2652 | -18.10 | <0.001 | |
| year | 0.5362 | 0.0645 | 8.32 | <0.001 | 71.6 |
| temporal autocorrelation > 0 | 0.8839 | 0.1439 | 6.14 | <0.001 | 35.0 |
| log(mean no. of cattle + 1) | 0.9826 | 0.1303 | 7.54 | <0.001 | 55.0 |
| log(mean spatial deer density + 0.01) | 0.5130 | 0.1810 | 2.83 | 0.005 | 6.3 |
| North-UTM | -1.2029 | 0.1804 | -6.67 | <0.001 | 41.5 |
| sqrt(distance to fjord) | -0.3908 | 0.1894 | -2.06 | 0.039 | 2.4 |
| Region "West" | 1.7398 | 0.2908 | 5.98 | <0.001 | 35.0 |

sqrt = square-root transformed; temporal autocorrelation = incidence of anaplasmosis previous year. Continuous variables are scaled to variance=1 and mean=0. NAO = North Atlantic Oscillation index. DJF = December-January-February. ΔAIC = the increase in AIC if removing the variable. Random effects are given by (A) 192 and 253 (B) municipalities. In A the municipalities are also nested in 10 counties. The change in AIC if removing the random intercepts is 495.4 (A) and 122.2 (B). If keeping only the year trend (and mean no. of cattle – B), ΔAIC is 26.5 (A) and 180.9 (B).

**Supplementary Table 4 | Model of anaplasmosis in sheep.** Parameter estimates of incidence from mixed-effects model using (A) counts in a negative binomial model and (B) presence or absence of disease in a logistic regression model of anaplasmosis in sheep from 10 counties in Norway from years 2000-2015.

| Parameter | Estimate | SE | z | p | ΔAIC |
|---|---|---|---|---|---|
| A. Negative binomial model | | | | | |
| Intercept | -13.5441 | 0.6747 | -20.07 | <0.001 | |
| year | 1.6184 | 0.1807 | 8.96 | <0.001 | 104.7 |
| year$^2$ | -0.6924 | 0.1297 | -5.34 | <0.001 | 26.8 |
| log(mean spatial deer density + 0.01) | 1.0101 | 0.2561 | 3.94 | <0.001 | 12.5 |
| No. of health recordings per producer | 0.7421 | 0.0911 | 8.15 | <0.001 | 73.2 |
| North-UTM | -1.8162 | 0.3505 | -5.18 | <0.001 | 28.6 |
| sqrt(prop(human settlement)) | -0.4454 | 0.2038 | -2.19 | 0.029 | 3.0 |
| Region «West» | 3.8411 | 0.6226 | 6.17 | <0.001 | 56.2 |
| B. Logistic regression model | | | | | |
| Intercept | -8.5376 | 0.7672 | -11.129 | <0.001 | |
| year | 0.6942 | 0.1066 | 6.510 | <0.001 | 45.1 |
| temporal autocorrelation > 0 | 0.6683 | 0.2253 | 2.967 | 0.003 | 6.6 |
| log(mean no. of sheep) | 1.648 | 0.240 | 6.861 | <0.001 | 53.5 |
| log(mean spatial deer density + 0.01) | 1.3483 | 0.3046 | 4.426 | <0.001 | 18.3 |
| No. of health recordings per producer | 0.8784 | 0.1331 | 6.599 | <0.001 | 50.5 |
| North-UTM | -2.4293 | 0.3973 | -6.114 | <0.001 | 42.8 |
| Region «West» | 3.4513 | 0.6302 | 5.476 | <0.001 | 39.1 |

sqrt = square-root transformed; prop = proportion; temporal autocorrelation = incidence of anaplasmosis previous year. Continuous variables are scaled to variance=1 and mean=0. NAO = North Atlantic Oscillation index. DJF = December-January-February. ΔAIC = the increase in AIC if removing the variable. Random effects are given by 188 (A) and 247 (B) municipalities. The change in AIC if removing the random intercepts is 249.9 (A) and 73.3 (B). If keeping only the year trend (and mean no. of sheep – B), ΔAIC is 138.6 (A) and 170.7 (B).

# Supplementary Discussion

### Potential biases in the disease records

Processes potentially creating bias may be linked to change in 1) diagnostic criteria, 2) reporting and/or 3) treatment.

**A. Lyme disease.** Lyme disease in humans is a reportable disease administered by the Norwegian Health authorities, the Norwegian. **1) Diagnostic criteria.** Diagnostic criteria have been consistent since 1995. In 2015, an increase in the age group 0-9 years may in part be linked to the use of spinal puncture becoming more standard way to diagnose the disease in children. Cases of simple Erythema migrans (EM) are not included in the MSIS statistics, only cases of systemic disease are included[1]. Therefore these statistics presented do not involve the majority of Lyme disease cases[2,3]. **2) Reporting rates**. Over the time period, there has been increased attention and awareness to Lyme disease. However, since these are more severe cases of the Lyme, they likely to have been reported more consistently through the study period. **3) Treatment.** There is no major change in the treatment of Lyme disease. Overall, these uncertainties should not affect the spatial pattern as Lyme disease records have an overall high quality[4].

**B. Babesiosis and anaplasmosis in cattle**. The national database on babesiosis and anaplasmosis in cattle covers over 90% of all dairy cattle in Norway[5]. Each individual dairy cow has a Cow Health Card which is registered on a database, where information with the veterinarian's diagnosis, description of the case and subsequent treatment are available. These data are part of a dairy recording system, which has been established nationwide since 1975[6]. **1a) Diagnostic of babesiosis**. Babesiosis is easily recognized with symptoms of bloody urine, which is pathognomonic with very few differential diagnoses. **1b) Diagnostic of anaplasmosis**. It is more difficult to diagnose anaplasmosis compared to both Lyme disease

and babesiosis. The bacterium *A. phagocytophilum* causing anaplasmosis attacks the white blood cells[7], therefore causing more general symptoms like high fever which will occur in many diseases. In anaplasmosis regularly causes immune depression leading to secondary diseases, and it could be difficult to identify anaplasmosis as the primary disease. However, the diagnostic criteria have not changed over the time. Anaplasmosis is therefore likely underreported, but this does not necessarily imply bias in terms of change over time. **2) Reporting rates**. The data quality for the major diseases, like mastitis, is considered very good with about 82% coverage in dairy cattle[8]. For disease in calves incidence is more under-reported with coverage of 40 % of the true incidence[9]. However, calf disease reporting has increased and improved during the study period from 1995 till 2016. From 2008 it was possible for veterinarians to record disease both in cattle and sheep by digitally directly into the databases. During 2016 about 90% of data was entered directly, digitally. Interestingly, as data coverage in calves and young stock improved, the incidence of babesiosis declined. Despite that the largest improvement of recording disease in calf and young stock was after 2005, these two diseases have been dropping in cattle after 2005. We believe the recording quality could not be the main reason for this initial increase and not at all for the subsequent decrease after 2005. **3) Treatment.** During the period 1995 to 2016 the recorded incidence of major diseases in dairy cattle has decreased by 60-70% due to intensive prevention and breeding program[6]. However, this is unlikely to be the case for rare disease like babesiosis and anaplasmosis. The typical treatment for anaplasmosis is the antibiotic Tetracycline, which is very seldom used in Norway. A general aim of reducing antibiotic use was initiated in 1995, and the total use of Tetracycline in Norway in dairy farming has declined by 70%. However, since babesiosis is not treated this way, the largely similar pattern of changes in this disease's incidence, suggests this reduction in overall use of antibiotic has not markedly affected the

specific treatment of anaplasmosis. Babesiosis is treated with imidacard, and the use of it is not registered in Norway.

**C. Anaplasmosis in sheep**. **1) Diagnostic criteria.** As for anaplasmosis in cattle, it is difficult to diagnose anaplasmosis in sheep; nevertheless the way it is diagnosed has not changed. **2) Reporting rates**. Most cases in the database likely come from the period of grazing infield during early spring, when lambs first get exposed to ticks. When sheep are grazing in the outfields during summer it is typically with limited surveillance in many cases. Though not all infected sheep contract anaplasmosis[10], the mortality of lambs may reach 20-30% in some flocks and survivors often suffer reduced body growth[11]. Hence, the total number of cases is likely markedly underreported. Further, there is no mandatory reporting of anaplasmosis in the sheep control in Norway. Hence, there is a higher likelihood of underreporting and bias compared to the other 3 disease records. The fact that we used the number of sheep in the database rather than total number of sheep in the municipality when calculating incidence will to some extent control for this. However, we cannot exclude the possibility of an increased level of recording of diseases as such, in the database over time. Therefore, we have entered overall reporting of diseases as a covariate in our analysis. **3) Treatment**. The way of treatment anaplasmosis in sheep has not changed over time.

## Supplementary References


1. Foreningen for utgivelse av Norsk legemiddelhåndbok http://legemiddelhandboka.no/Terapi/683. (2013).
2. Eliassen, K., Berild, D., Reiso, H., et al. Incidence and antibiotic treatment of erythema migrans in Norway 2005-2009. *Ticks Tick Borne Dis* **8**, 1-8 (2017).
3. MacDonald, E., Vestrheim, D. F., White, R. A., et al. Are the current notification criteria for Lyme borreliosis in Norway suitable? Results of an evaluation of Lyme borreliosis surveillance in Norway, 1995–2013. *BMC Public Health* **16**, 1-11 (2016).
4. Mysterud, A., Easterday, W. R., Stigum, V. M., Aas, A. B., Meisingset, E. L. & Viljugrein, H. Contrasting emergence of Lyme disease across ecosystems. *Nature Comm* **7**, Article 11882(2016).
5. Jore, S., Viljugrein, H., Hofshagen, M., et al. Multi-source analysis reveals latitudinal and altitudinal shifts in range of *Ixodes ricinus* at its northern distribution limit. *Parasite Vector* **4**, 84(2011).
6. Østerås, O., Solbu, H., Refsdal, A. O., Roalkvam, T., Filseth, O. & Minsaas, A. Results and evaluation of thirty years of health recordings in the Norwegian dairy cattle population. *J Dairy Sci* **90**, 4483-4497 (2007).
7. Woldehiwet, Z. The natural history of *Anaplasma phagocytophilum*. *Vet Parasitol* **167**, 108-122 (2010).
8. Wolff, C., Espetvedt, M., Lind, A. K., et al. Completeness of the disease recording systems for dairy cows in Denmark, Finland, Norway and Sweden with special reference to clinical mastitis. *BMC Veterinary Research* **8**, article 131(2012).
9. Gulliksen, S. M., Lie, K. I. & Østerås, O. Calf health monitoring in Norwegian dairy herds. *J Dairy Sci* **92**, 1660-1669 (2009).

10. Stuen, S. & Bergström, K. Serological investigation of granulocytic Ehrlichia infection in sheep in Norway. *Acta Vet Scand* **42**, 331-338 (2001).

11. Grøva, L., Olesen, I., Steinshamn, H. & Stuen, S. Prevalence of *Anaplasma phagocytophilium* infection and effect on lamb growth. *Acta Vet Scand* **53**, 30(2011).
